# Assessing knee joint biomechanics and trunk posture according to medial osteoarthritis severity

**DOI:** 10.1038/s41598-023-46486-1

**Published:** 2023-11-06

**Authors:** Yuki Suzuki, Yasumitsu Ohkoshi, Kensaku Kawakami, Kenta Shimizu, Shuya Chida, Kengo Ukishiro, Tomohiro Onodera, Koji Iwasaki, Tatsunori Maeda, Sho’ji Suzuki, Eiji Kondo, Norimasa Iwasaki

**Affiliations:** 1 Department of Orthopaedic Surgery, Faculty of Medicine and Graduate School of Medicine, Hokkaido University, Kita 15 jo, Nishi 7 chome, Kita-ku, Sapporo, Hokkaido 060-8638 Japan; 2 Department of Orthopedic Surgery, Hakodate Orthopedic Clinic, Ishikawa-cho2-115, Hakodate, Hokkaido 041-0802 Japan; 3 Department of Production Systems Eng., National Institute of Technology, Hakodate College, Tokura-cho 14-1, Hakodate, Hokkaido 042-8501 Japan; 4 Department of Rehabilitation, Hakodate Orthopedic Clinic, Ishikawa-cho2-115, Hakodate, Hokkaido 041-0802 Japan; 5 Department of Functional Reconstruction for the Knee Joint, Graduate School of Medicine, Hokkaido University, Kita 15 jo, Nishi 7 chome, Kita-ku, Sapporo, Hokkaido 060-8638 Japan; 6 Department of Complex and Intelligent Systems, Future University Hakodate, Kamedanakano-cho 116-2, Hakodate, Hokkaido 041-8655 Japan; 7 Centre for Sports Medicine, Hokkaido University Hospital, Kita 14 jo Nishi 5 chome, Kita-ku, Sapporo, Hokkaido 060-8648 Japan

**Keywords:** Bone quality and biomechanics, Orthopaedics

## Abstract

During progression of knee osteoarthritis (OA), gait biomechanics changes three-dimensionally; however, its characteristics and trunk posture according to OA severity remain unknown. The present study investigated three-dimensional knee joint biomechanics and trunk posture according to knee OA severity. Overall, 75 patients (93 knees) with medial knee OA [Kellgren-Lawrence grade ≥ 2, grade 2: 20 patients with 24 knees (mean 60.0 years old); grade 3: 25 with 28 knees (mean 62.0 years old); grade 4: 30 with 41 knees (mean 67.9 years old)] and 14 healthy controls (23 knees, mean 63.6 years old) underwent gait analysis using an optical motion capture system and point cluster technique. In grade 2 knee OA, the relative contribution of the knee adduction moment (KAM) increased significantly (*P* < 0.05), and that of the knee flexion moment decreased (*P* < 0.05) prior to significant progression of varus knee deformity. Grade 3 knee OA showed significant exacerbation of varus knee deformity (*P* < 0.01) and KAM increase (*P* < 0.001). The maximum knee extension angle decreased (*P* < 0.05) and trunk flexion increased during gait in grade 4 knee OA (*P* < 0.001). Our study clarified the kinematics and kinetics of medial knee OA with trunk flexion according to severity. Kinetic conversion occurred in grade 2 knees prior to progression of varus deformities, knee flexion contractures, and sagittal imbalance during gait in patients with severe knee OA.

## Introduction

Knee osteoarthritis (OA) is a degenerative disease affecting more than 300 million patients worldwide. In severe cases, dysfunction, malalignment, joint stiffness, deformity, and flexion contracture occur. This leads to chronic pain, reduced activities of daily living, and disability, especially in older adults^1–3^. The knee joint is responsible for one-joint motion that enables whole-body movement by interlocking with other joints and rotation of the trunk. Maintaining a standing posture is achieved through the interaction and compensatory mechanisms of the knee motion, spinal column, and pelvis. When the compensatory function of pelvic retroversion reaches a maximum, due to limited hip joint extension, knee flexion is mobilized^4^. This pathology, a consequence of progression of knee OA, is known to cause frailty and affects healthy life expectancy^1,5^.

Knee biomechanics is important for understanding knee OA pathology. External knee joint moment can be divided into the knee adduction moment (KAM), knee flexion moment (KFM), and knee rotation moment (KRM), which are important concepts in understanding the mechanical environment of the knee joint^6,7^. Many studies have been conducted with gait analysis in patients with knee OA^8–10^. Moreover, an increased KAM due to varus alignment has been associated with rapid progression of knee OA^6,7,10,11^. Asay et al. also assessed the relationship between knee OA progression and changes in gait after 5 years in 19 patients with medial knee OA^12^. The results indicated that as knee OA progressed, the relative contribution of KAM to the total joint moment (TJM) (the new concept in kinetics) increased and KFM decreased. However, knee biomechanics according to the severity of knee OA remains unknown.

Recently, it has been reported that not only changes in coronal parameters but also variations in sagittal and axial parameters occurred in the kinematics and kinetics of knee OA. These parameters have been used for gait analysis for a better understanding of knee OA^13,14^. Additionally, external knee joint moments were found to be strongly influenced by the position of the center of the body’s weight^15,16^. Therefore, analysis of trunk posture is necessary to understand three-dimensional joint dynamics. Determining the onset and cause of knee moment imbalance, knee flexion contracture, and trunk flexion are critical for devising systematic treatment strategies for the knee joint and spine. However, regarding comprehensive understanding of knee OA pathology, the three-dimensional knee joint biomechanics and trunk posture according to knee OA severity remains unknown.

This study aimed to investigate the three-dimensional knee joint biomechanics and trunk posture according to knee OA severity. We hypothesized that the onset of knee moment imbalance, knee flexion contracture, and trunk flexion depends on OA severity.

## Materials and methods

This retrospective study was conducted in accordance with our institutional ethics policy. The institutional review board (Ethics committee of Hakodate Orthopedics Clinic) approved this study (No. HOC-C01). The procedures followed were in accordance with the ethical standards of the responsible committee on human experimentation (institutional and national) and with the Helsinki Declaration of 1975, as revised in 2000. Informed written consent was obtained from all patients for providing patient information and any accompanying supplements, and for publication.

### Participants

**Figure 1:**
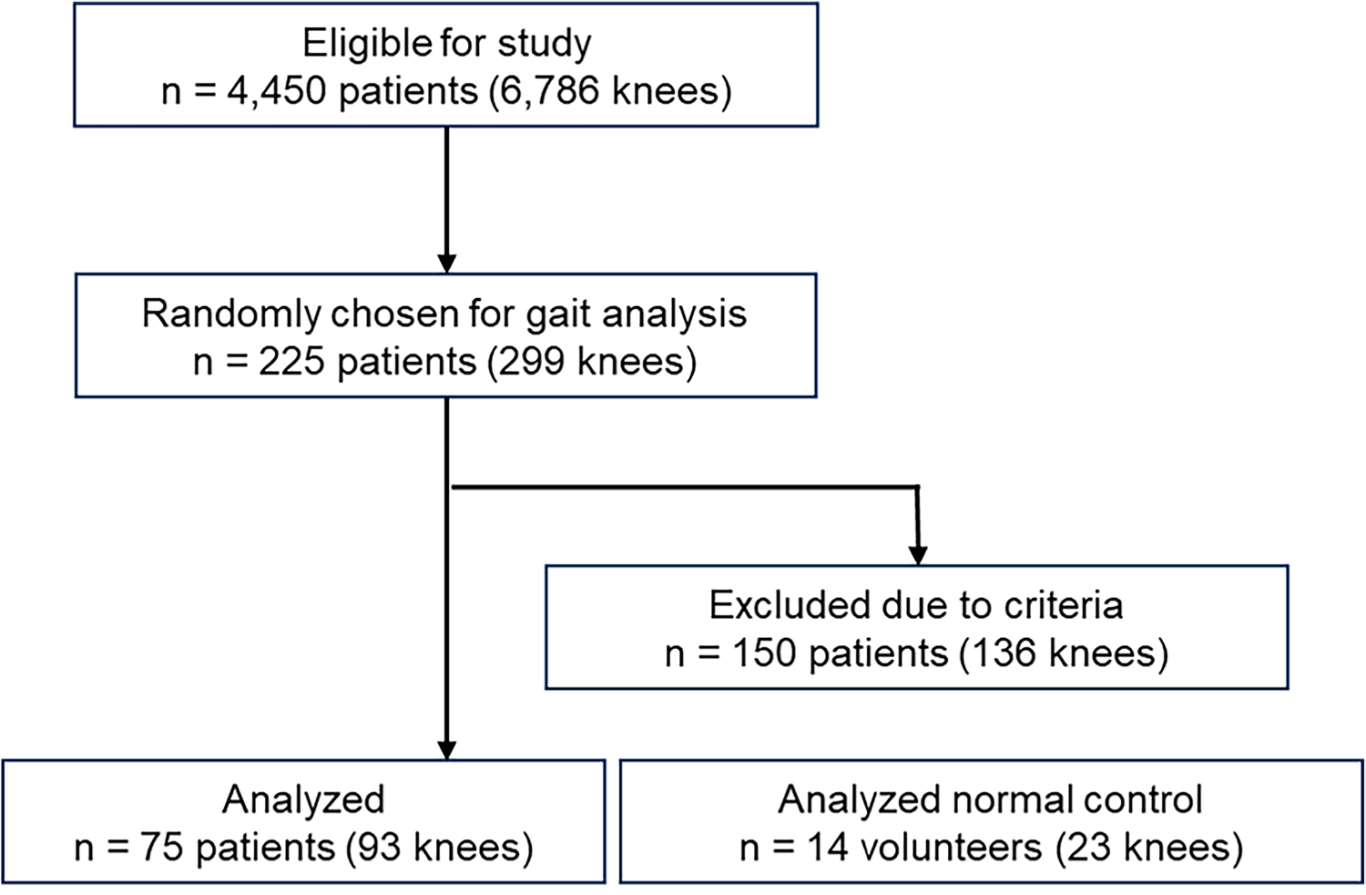
Flowchart of the present study.

From November 2014 to October 2018, a total of 4450 patients (6786 knees) were diagnosed with knee OA at our medical facility. Patients aged over 40 years, radiographically diagnosed with medial compartment knee OA with Kellgren–Lawrence (K-L) grade ≥ 2 in at least one knee, and who were randomly chosen to undergo gait analysis were enrolled in the study (Fig. 1). Patients with OA of the hip or ankle, disability preventing walking without support, predominantly lateral compartment OA, neurological deficits, or previous spinal fractures were excluded from the study, as were patients who had undergone orthopedic surgery. Thereafter, a total of 75 patients (93 knees) were eligible for participation in the study, including 20 patients with 24 grade 2 knee OA (group G-2), 25 with 28 grade 3 knee OA (group G-3), and 30 with 41 grade 4 knee OA (group G-4) in the OA groups. As normal controls, 14 healthy volunteers (23 knees, aged 52–69 years), including three male and eleven female participants, were allocated to group C.

### Physical and radiological evaluation

Knee range of motion (ROM) was measured for physical evaluation. Radiologically, the K-L classification was used to assess knee OA severity^17^. Radiological evaluation was performed using anteroposterior radiographs. Radiographic parameters included the hip-knee-ankle angle (HKA) in full length bilateral standing anteroposterior radiographs and the posterior tibial slope angle in lateral view.

### Gait analysis

The participants performed three sets of overground walking trials at their self-selected normal walking speeds. Gait data were collected as described previously^12,18,19^. Gait motion data were captured using a three-dimensional motion analysis system with eight infrared light cameras (ProReflex Qualisys AB Inc., Gothenburg, Sweden). Ground reaction force was measured in three dimensions in the global coordinate system using two multi-component force plates (OR6, Advanced Mechanical Technology Inc., Watertown, NY, USA) embedded in an 8-m walkway. Motion and force data were synchronized and collected at 120 Hz. Three-dimensional motion data were processed using Qualisys Track Manager (Qualisys Track Manager 3D; Qualisys AB Inc., Gothenburg, Sweden). Force data were used to identify the heel strike times in each gait cycle. The gait cycle was defined as the interval between heel strikes on the ipsilateral side.

Kinematic data were collected using the point cluster technique, as previously described^20^. Fifty-eight light-reflective markers with 14 mm diameter were placed by physical therapists well-trained in localizing the anatomical landmarks, and were used to calculate knee motion in six degrees of freedom with no constraints, as previously described^21^. Fifty-six light-reflective markers with 14 mm diameter were arranged on two limb segments, creating separate clusters of thirteen markers on the thigh and fifteen on the shank^22^. The software application BioMove (Stanford University, Stanford, CA, USA) was used to calculate the joint kinematics and trunk flexion during walking. External joint moments were calculated using standard inverse dynamics and normalized to each participant’s body weight and height^23^. The angle of trunk flexion was calculated as the angle between the vertical axis and the line segment connecting the midpoints of both acromion markers and obliques in the sagittal plane of the global coordinate system^24^. Each gait cycle was time-normalized to 100% of the gait cycle, where the initial contact of a limb inside a force plate was defined as 0%, and the following ground contact of the same limb was defined as 100%.

The values extracted from three walking trials were averaged. The extracted moments included KAM, KFM, and KRM. Similar to the knee index, the TJM (TJM = √ (KAM^2^ + KFM^2^ + KRM^2^) was calculated during each frame of the stance phase by taking the square root of the sum of the squares of the KFM, KAM, and KRM. The first peak of the TJM (TJM1) was defined as the maximum TJM during the first half of the stance, and the second peak TJM (TJM2) was defined as the maximum TJM during the second half of the stance. At the time of the TJM peaks, the proportions of the KFM (%KFM), KAM (%KAM), and KRM (%KRM) contributions to the total joint moment were calculated as the square of the planar moment at the time of the peak over the square of the total moment at the time of the peak, multiplied by 100 (%KAM = KAM^2^/TJM^2^*100)^12^.

### Statistical analysis

Comparison of the patients’ demographic data, joint moments, and kinetics among each K-L grade, and those of the normal controls, were assessed by one-way analysis of variance, followed by the Bonferroni test. All analyses were performed with JMP software (SAS, Cary, NC, USA). *P* values < 0.05 were considered statistically significant. Since the sample size required to obtain α = 0.05 and power = 0.8 for four groups is 76, we concluded that the number of cases (89 patients 116 knees in total) is sufficient for statistical consideration. All the figures were done by Adobe Illustrator CC 2020 (Adobe, CA, USA).

## Results

### Demographic and radiographic results

The demographic and radiographic data of the participants are shown in Table 1. There was no significant difference in the mean age, and in proportion of males and females among groups. The height, weight and body mass index were low in group C. In group G-4, gait speed was significantly slower, and the HKA was significantly smaller. Knee flexion, extension angle, and total range of motion were significantly smaller in group G-4. No significant differences were observed in the posterior tibial slope angle.

**Table 1:** Background and radiographic data of normal controls and patients in each Kellgren–Lawrence grade.

	Group C	Group G-2	Group G-3	Group G-4
Number of participants (patients (knees))	14 (23)	20 (24)	25 (28)	30 (43)
Age (years)	63.6 ± 5.2	60.0 ± 8.1	62.0 ± 8.2	67.9 ± 10.6
Sex (Male (knees): Female (knees))	3 (4): 11 (19)	10 (11): 10 (13)	11(11): 14 (17)	6 (9): 24 (34)
Height (cm)	152.0 ± 6.1	161.1 ± 7.2^b^	159.8 ± 9.8^b^	155.2 ± 9.2^b^
Weight (kg)	50.3 ± 11.0	66.4 ± 11.0^b^	64.7 ± 9.5^b^	65.4 ± 11.3^b^
Body mass index (kg/cm^2^)	21.6 ± 3.7	25.5 ± 3.4^b^	25.3 ± 2.6^b^	27.2 ± 4.3^b^
Gait speed (m/s)	1.3 ± 0.2	1.2 ± 0.2^a^	1.1 ± 0.2^b^	1.0 ± 0.2^b,e^
Knee maximum flexion angle (°)	151.7 ± 5.6	145.0 ± 5.5^b^	143.0 ± 8.5^b^	134.9 ± 13.3^b,d,e^
Knee maximum extension angle (°)	0.2 ± 1.0	− 3.1 ± 5.3^a^	− 3.4 ± 5.6^a^	− 7.3 ± 5.1^b,c,e^
Knee range of motion (°)	151.5 ± 5.5	148.1 ± 6.4	146.4 ± 6.8^a^	141.7 ± 10.6^b,c^
Hip–knee–ankle angle (°)	− 1.3 ± 2.5	− 3.4 ± 2.2^a^	− 4.5 ± 2.7^b^	− 8.4 ± 3.2^b,e,f^
Percentage of mechanical axis (%)	42.0 ± 11.0	32.9 ± 9.6^a^	28.7 ± 10.9^b^	12.4 ± 13.8^b,e,f^
Posterior tibia slope angle (°)	9.4 ± 2.4	8.4 ± 3.6	8.4 ± 3.0	9.0 ± 3.2

^a^Significantly different from group C (*P* < 0.05). ^b^Significantly different from group C (*P* < 0.01). ^c^Significantly different from group G-2 (*P* < 0.05). ^d^Significantly different from group G-3 (*P* < 0.05). ^e^Significantly different from group G-2 (*P* < 0.01). ^f^Significantly different from group G-3 (*P* < 0.01).

### Kinetics

#### External knee moments

**Figure 2:**
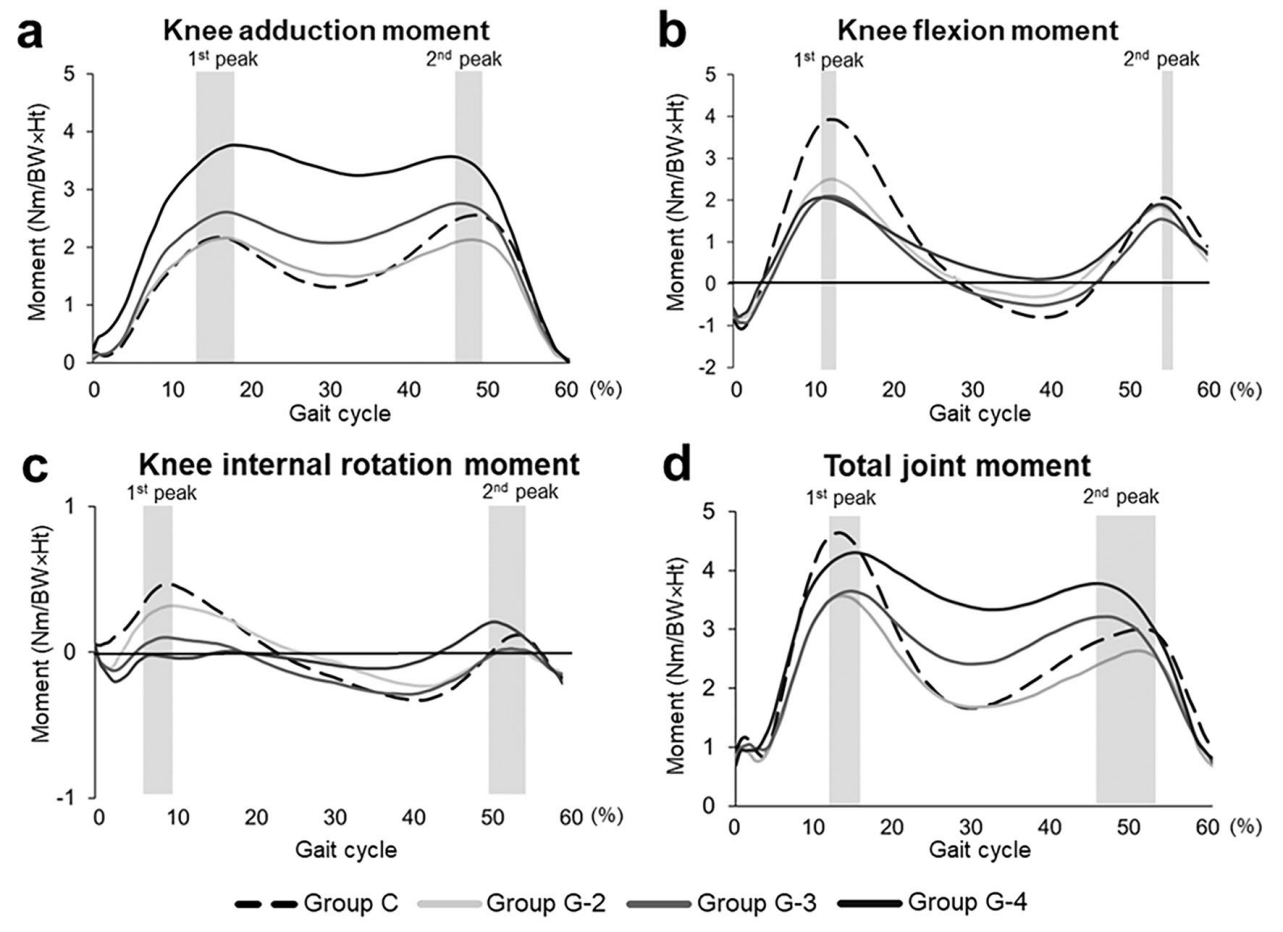
External knee moment and total joint moment (TJM) in each osteoarthritis grade and control participants. (**a**) Knee adduction moment (KAM). Group C versus Groups G-4 at the first peak (*P* < 0.01), Groups G-2,-3 versus Group G-4 at the first peak (*P* < 0.01), Groups C and G-2 versus Group G-4 at the second peak (*P* < 0.01). (**b**) Knee flexion moment (KFM). Group C versus Groups G-2 (*P* < 0.05), -3 (*P* < 0.01), and -4 (*P* < 0.01) at the first peak, Group C versus Groups G-4 at the bottom in the midstance phase (*P* < 0.01). (**c**) Internal rotation moment of the knee. Group G-2 versus Group G-4 at the first peak (*P* < 0.05), Group C versus Groups G-3,-4 at the first peak (*P* < 0.01). (**d**) TJM. Group C versus Group G-2 at the first peak (*P* < 0.05), among all groups in Group G-2 (*P* < 0.05),-3 (*P* < 0.01), and -4 (*P* < 0.01) at the bottom in the midstance phase, and Group G-4 versus Groups C (*P* < 0.05) and G-2 at the second peak (*P* < 0.01).

The KAM showed bimodal peak patterns in the early and late stance phases in all groups (Fig. 2a). At the first peak of the adduction moment (KAM1), the KAM was higher in group G-4 (*P* < 0.001) than that in group C. Furthermore, the KAM was higher in group G-4 than in groups G-2 (*P* < 0.001) and G-3 (*P* = 0.0052). At the second peak of the adduction moment (KAM2), the KAM was higher in group G-4 than in groups C (*P* < 0.001), G-2 (*P* < 0.001).

For the KFM, all group showed bimodal peak patterns in the early and late stance phases in (Fig. 2b). Group C showed a moment peak during the loading response phase, which then decreased and was converted into an extension moment in the midstance phase, followed by a second peak in the late stance phase. The KFM was low in the OA groups compared with group C—with significant differences between group C and group G-2 (*P* = 0.013), G-3 (*P* < 0.001), and G-4 (*P* < 0.001) at the first peak (KFM1). However, the KFM showed no significant difference among the OA groups at both KFM1 and the second peak (KFM2). The KFM2 was higher in group G-3 (*P* = 0.046) than that in group C. A decrease in KFM was observed, and had a negative value, in groups C, G-2, and G-3 in the midstance phase (from a peak at 12–13% and reached a minimum at approximately 38% of the gait phase). However, translation of the KFM into an extension moment was not observed in group G-4. The KFM was significantly higher in group G-4 than in group C (*P* = 0.034).

The KRM showed a bimodal peak pattern in groups C, G-2, and G-3, but not in group G-4, which only showed a peak in the late stance phase (Fig. 2c). The KRM showed a transition from a positive value (internal rotation) in the early stance phase to a negative value (external rotation) in the late stance phase. The KRM was significantly lower in the severe groups (group C versus groups G-3 (*P* < 0.001) and G-4 (*P* < 0.001) in the early stance phase).

#### TJM and the relative contributions of each moment component

The TJM showed a bimodal peak pattern throughout the stance phase in all groups (Fig. 2d). The TJM was significantly higher in group C than in group G-2 (*P* = 0.035) at the first peak (TJM1). The TJM was remarkably higher in group G-4 than in groups C (*P* = 0.027) and G-2 (*P* < 0.001) at the second peak (TJM2).

**Figure 3:**
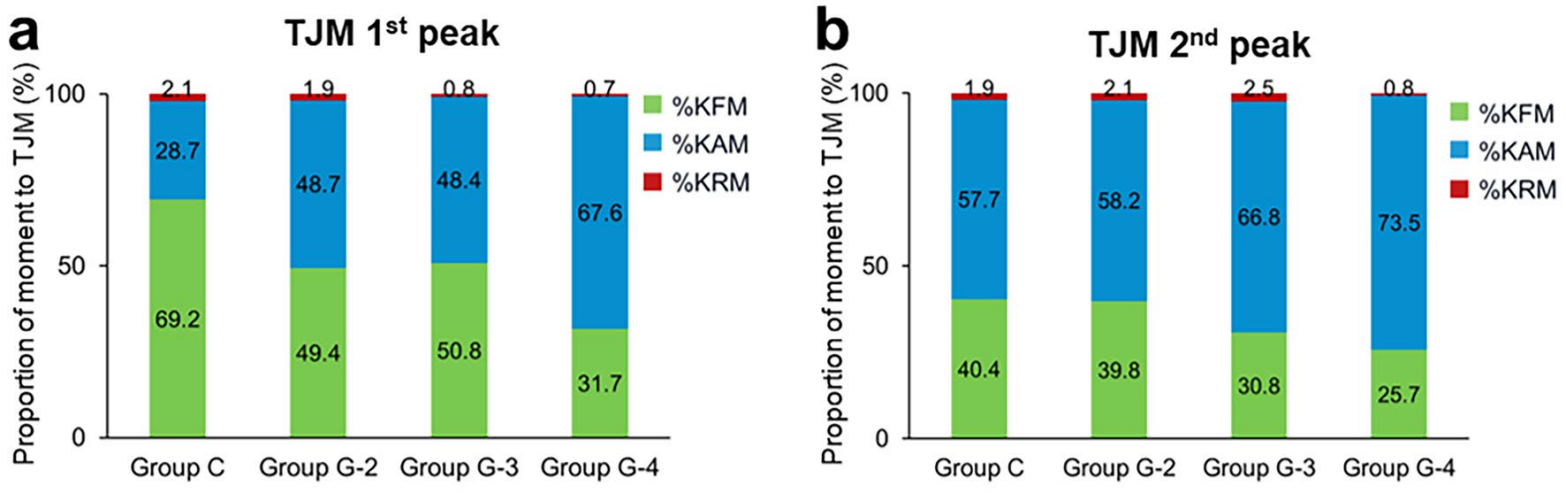
Changes in the relative contributions of each external moment component at the knee. (**a**) The relative contribution of each component according to osteoarthritis grade and control participants at the first peak of the total joint moment (TJM). The knee adduction moment (%KAM), flexion moment (%KFM), and rotation moment (%KRM). Group C versus Groups G-2 (*P* < 0.05: %KAM&%KFM), Group C versus Groups G-3, (*P* < 0.01: %KAM&%KFM), Groups G-4 (*P* < 0.01: %KAM&%KFM&%KRM), Groups G-2,-3 versus Group G-4 in %KAM (*P* < 0.05). (**b**) The relative contribution of each component to the second peak of the TJM. There was no significant difference among groups.

Regarding the relative contribution, the normal controls showed a high %KFM, followed by %KAM and %KRM at TJM1. In group G-4 at TJM1, however, the %KAM was significantly high (*P* < 0.001) and the %KFM was significantly low (*P* < 0.001) (Fig. 3a). In group C at TJM1, %KAM was lower and %KFM was higher than in other groups (*P* < 0.05). At TJM2, all groups showed a high %KAM, followed by %KFM and %KRM (Fig. 3b). Group G-4 showed the same tendency of high %KAM and low %KFM, but there were no significant differences.

### Kinematics

**Figure 4:**
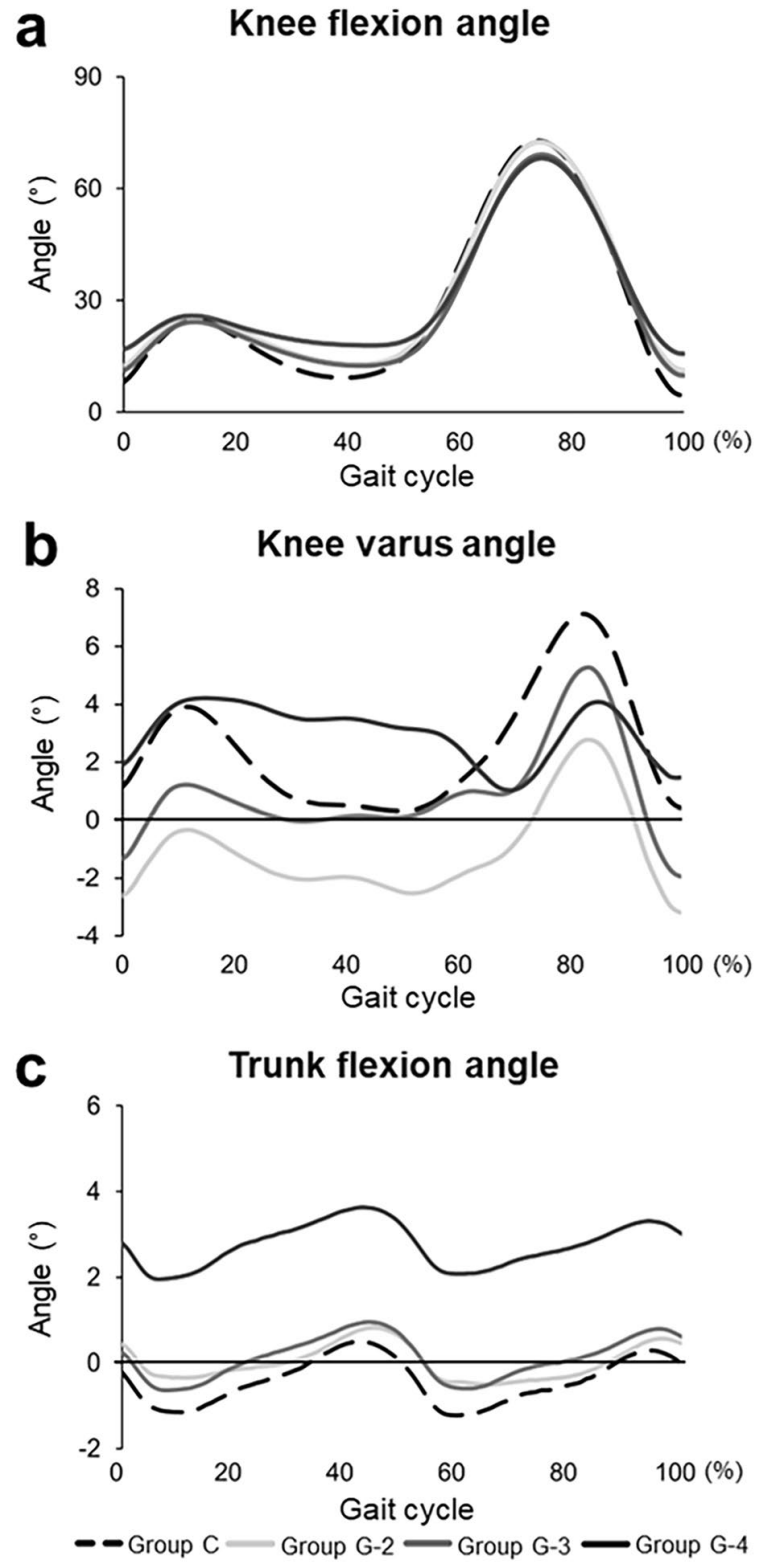
Kinematics of the knee and trunk flexion in each osteoarthritis grade and control participants. (**a**) Knee flexion angle. Initial contact: Group C versus Groups G-4 (*P* < 0.05), Group G-2,-3 versus Group G-4 (*P* < 0.01). Bottom in the midstance phase: Group C versus Group G-2 (*P* < 0.05) and G-4 (*P* < 0.01), Group G-2,-3 versus Group G-4 (*P* < 0.01). Second peak: Group C versus Groups G-3,-4 (*P* < 0.05). (**b**) Knee varus angle. Groups G-4 versus Group G-2 (*P* < 0.01) and G-3 (*P* < 0.05) in the stance phase. Group C versus Group G-2 (*P* < 0.05) and G-4 (*P* < 0.05) in the stance phase. (**c**) Trunk flexion angle. Group C, G-2, and G-3 versus Group G-4, throughout the gait cycle (*P* < 0.01).

The knee flexion angle showed a similar pattern during the gait cycle in all the groups. Group C showed a smaller knee flexion angle than that of group G-4 at initial contact (*P* = 0.015) and midstance phase (*P* < 0.01), and a larger flexion angle than that of group G-3 (*P* = 0.020) and G-4 (*P* = 0.018) at the second peak (Fig. 4a). Group G-4 showed a larger knee flexion than that of group G-2 (*P* < 0.01) and G-3 (*P* < 0.01) at initial contact, mid- to terminal stance, and swing phase. Changes in the knee flexion angle were significantly lower in group G-4 than in other groups from initial contact to first peak (*P* < 0.01) and from first peak to midstance (*P* < 0.05). The knee varus angle was significantly higher in group G-4 than in the other groups (*P* < 0.05) from mid- to terminal stance phase (Fig. 4b).

### Trunk flexion

Trunk flexion showed a negative peak in the early stance phase and a positive peak in the late stance phase in all groups (Fig. 4c). The late stance phase (42–45% of the stance phase) was the same as the maximum knee extension phase (39–45% of the stance phase) in the midstance phase. Trunk flexion significantly increased in patients with grade 4 knee OA, but not in those of the other groups, throughout the gait cycle (*P* < 0.01).

## Discussion

In this study, the severe knee OA group showed more severe varus deformites, a larger KAM, and a smaller KFM. The relative contribution converted from %KFM dominance into %KAM dominance in patients with severe knee OA at TJM1. In addition, knee flexion contracture and trunk flexion were observed in group G-4. We evaluated knee alignment, ROM, and trunk flexion, and clarified the pathogenesis and mechanism of OA progression from a biomechanical perspective regarding kinematic and kinetic characteristics in each group. This is the first study to identify differences in kinematics and kinetics, including the relative contributions of each moment component, according to OA severity.

Regarding varus deformity and the KAM, significant varus deformity and increases in the KAM occurred in groups G-3 and G-4 (Table 1, Fig. 2). This may be the result of increases in the lever arm of the KAM as well as the severity of the varus deformities. However, there was a significant increase and decrease in the relative contributions of KAM and KFM, respectively, in grade 2 knees, with little difference between grade 2 and 3 knee OA (Fig. 3). This suggests that the conversion of the relative contribution of each moment from KAM to KFM had already occurred in grade 2 knee OA prior to varus deformity appearance.

Group G-4 was found to have a significantly higher %KAM, and lower %KFM, compared with groups G-2 and G-3, showing further drastic kinetic changes in severe knee OA (Fig. 3). Additionally, significant flexion contractures and trunk flexion were observed, indicating that grade 4 differed from grade 3 knee OA (Table 1, Fig. 4). In addition to varus deformities, severe knee OA was reported to have an of apparent sagittal imbalance^25–27^. Ohkoshi et al. reported that trunk flexion, which leads to elongation of the moment arm of the trunk toward the center of gravity, reduced quadriceps muscle activity in the squat movement^28^. The same effect, of reduction in KFM, may occur during gait. Our results suggest that grade 4 knee OA has the potential to increase not only the risk of quadriceps muscle weakness^29^ and patellofemoral arthropathy exacerbation^30^ due to knee flexion contracture, but also the risk of erector spinae muscle weakness^31,32^ and vertebral fractures. Our data support and explain the mechanism of vertebral fractures associated with knee OA in a previous biomechanical study^33^.

**Figure 5:**
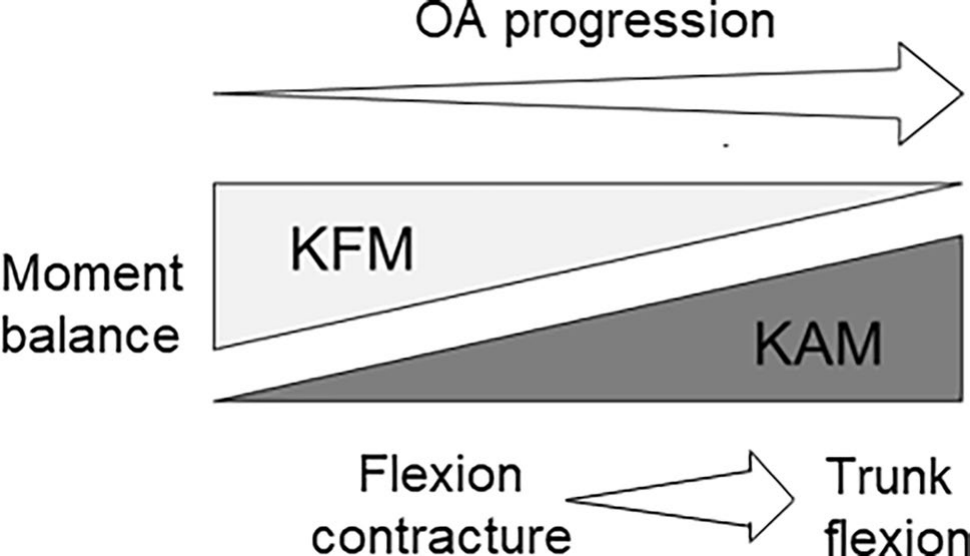
Scheme of severity of knee osteoarthritis (OA) and its pathophysiology. Knee OA progression leads to a conversion of the knee flexion moment (KFM) to the knee adduction moment (KAM). This alteration in moment balance subsequently exacerbates knee flexion contracture during gait, eventually leading to trunk flexion.

The results of this study showed that a change in the relative moment contribution occurred in grade 2 knee OA, with significant constructive varus deformity in grade 3 (Table 1, Fig. 3). Knee OA progression leads to a conversion of the KFM to the KAM. This change in moment balance subsequently exacerbates knee flexion contracture during gait, resulting in trunk flexion (Fig. 5). These outcomes are clinically relevant, suggesting that conservative treatment, such as orthotic treatment^34^ and muscle strength training^35^, may be particularly effective in grade 2 knee OA before constructive changes occur. This is consistent with the results of previous studies of therapeutic interventions with rehabilitation^36,37^. It has been reported that quadriceps strength weakens with age and pain even in mild cases of knee OA^37^. In contrast, in patients with grade 3 OA, prevention of flexion contracture and maintenance of quadriceps muscle strength are important to prevent progression to grade 4. This may indicate the need for surgical intervention (alignment surgery) to correct constructive varus deformities. In addition, patients with grade 4 knee OA had trunk flexion, which also affected sagittal imbalance. Therefore, more aggressive surgical treatment of grade 4 knee OA should be considered to improve overall function and prevent frailty.

The study has several limitations. First, the study included a relatively small number of participants and sex difference was not evaluated. The purpose of this study was to clarify the pathophysiology of knee OA by subdividing patients with knee OA and healthy participants into grades. Thus, the number of cases in each group was small. However, new findings were obtained with this grouping, and the study is considered reasonable and relevant. Furthermore, considering the pathophysiology of knee OA, the findings of the present study might be significant as it is a comparative study of grades of knee OA with healthy participants as controls. We also wanted to highlight that there were no significant differences in the proportion of males and females among all groups, which aligns with previous research^38^, making these group compositions reasonable for evaluating natural pathology of knee OA. Second, the measurement of kinematics and kinetics may have potential error. The point cluster technique has potential errors within 3 mm and 4°^39^. However, the accuracy of inverse dynamics is not known because the true value cannot be precisely evaluated. Values calculated by inverse dynamics may include potential errors due to adapting estimated values from previous studies in the process of calculation, such as those of segment gravity and quantity ratio^40^. However, these methods have been widely used in previous studies for gait analysis and these potential errors should be taken into account when interpreting gait kinetics data. Third, we used self-selected walking-speed for gait analysis. Generally, it is well known that gait parameters change according to walking speed. However, controlling walking speed may change the characteristics of knee OA gait. The gait speed was slow in OA groups, which might be resulted from knee pain and reflect the OA pathology. To assess the natural history and pathology of knee OA, we chose to use self-selected normal walking speeds, as in previous studies^41,42^. The enrolled controls were age-matched and ideal for comparing OA and non-OA. Finally, our data are cross-sectional, and whether the result is primary or secondary to disease status is not known. The course of the disease is also unclear. However, it is feasible to compare the characteristics of each grade at the same time. Variations in disease status among grades may be investigated in another study, but our study mainly targeted differences among grades and had an advantage on this point. This new concept, comparing changes in kinetics, including the relative contribution of moments, and kinematics data simultaneously among groups with different severities provides new insights into OA knee pathogenesis.

In conclusion, the present study assessed kinematics and kinetics according to the severity of medial knee OA with trunk flexion. The relative contribution of each moment component changed from KFM to KAM dominance, and flexion contracture and trunk flexion was observed in patients with severe knee OA. Kinetic conversion occurred in grade 2 knees prior to progression of varus deformities, knee flexion contractures, and sagittal imbalance during gait in patients with severe knee OA.

## Data Availability

The datasets used and analyzed during the current study are available from the corresponding author on reasonable request.

## References

[1] Abbott JH, Usiskin IM, Wilson R, Hansen P, Losina E (2017). The quality-of-life burden of knee osteoarthritis in New Zealand adults: A model-based evaluation. PLoS ONE.

[2] Campbell TM, McGonagle D (2021). Flexion contracture is a risk factor for knee osteoarthritis incidence, progression and earlier arthroplasty: Data from the Osteoarthritis Initiative. Ann. Phys. Rehabil. Med..

[3] Felson DT (2006). Clinical practice. Osteoarthritis of the knee. N. Engl. J. Med..

[4] Roussouly P, Pinheiro-Franco JL (2011). Biomechanical analysis of the spino-pelvic organization and adaptation in pathology. Eur. Spine J..

[5] Ohsawa T (2016). Relationship between knee osteoarthritis and the locomotive syndrome risk tests: A cross-sectional study. J. Orthop. Sci..

[6] Chang AH (2015). External knee adduction and flexion moments during gait and medial tibiofemoral disease progression in knee osteoarthritis. Osteoarthr. Cartil..

[7] Miyazaki T (2002). Dynamic load at baseline can predict radiographic disease progression in medial compartment knee osteoarthritis. Ann. Rheum. Dis..

[8] Na A, Piva SR, Buchanan TS (2018). Influences of knee osteoarthritis and walking difficulty on knee kinematics and kinetics. Gait Posture.

[9] Hamai S (2009). Knee kinematics in medial osteoarthritis during in vivo weight-bearing activities. J. Orthop. Res..

[10] Chehab EF, Favre J, Erhart-Hledik JC, Andriacchi TP (2014). Baseline knee adduction and flexion moments during walking are both associated with 5 year cartilage changes in patients with medial knee osteoarthritis. Osteoarthr. Cartil..

[11] Sharma L (2001). The role of knee alignment in disease progression and functional decline in knee osteoarthritis. JAMA.

[12] Asay JL, Erhart-Hledik JC, Andriacchi TP (2018). Changes in the total knee joint moment in patients with medial compartment knee osteoarthritis over 5 years. J. Orthop. Res..

[13] Favre J, Erhart-Hledik JC, Andriacchi TP (2014). Age-related differences in sagittal-plane knee function at heel-strike of walking are increased in osteoarthritic patients. Osteoarthr. Cartil..

[14] Meireles S (2017). Medial knee loading is altered in subjects with early osteoarthritis during gait but not during step-up-and-over task. PLoS ONE.

[15] Li K (2013). Trunk muscle action compensates for reduced quadriceps force during walking after total knee arthroplasty. Gait Posture.

[16] Simic M, Hunt MA, Bennell KL, Hinman RS, Wrigley TV (2012). Trunk lean gait modification and knee joint load in people with medial knee osteoarthritis: the effect of varying trunk lean angles. Arthritis Care Res. (Hoboken).

[17] Kellgren JH, Lawrence JS (1957). Radiological assessment of osteo-arthrosis. Ann. Rheum. Dis..

[18] Ino T (2015). Side-to-side differences of three-dimensional knee kinematics during walking by normal subjects. J. Phys. Ther. Sci..

[19] Miura K (2020). Kinematics and center of axial rotation during walking after medial pivot type total knee arthroplasty. J. Exp. Orthop..

[20] Dyrby CO, Andriacchi TP (2004). Secondary motions of the knee during weight bearing and non-weight bearing activities. J. Orthop. Res..

[21] Grood ES, Suntay WJ (1983). A joint coordinate system for the clinical description of three-dimensional motions: Application to the knee. J. Biomech. Eng..

[22] Andriacchi TP, Alexander EJ, Toney MK, Dyrby C, Sum J (1998). A point cluster method for in vivo motion analysis: Applied to a study of knee kinematics. J. Biomech. Eng..

[23] Chehab EF, Andriacchi TP, Favre J (2017). Speed, age, sex, and body mass index provide a rigorous basis for comparing the kinematic and kinetic profiles of the lower extremity during walking. J. Biomech..

[24] Hunt MA (2008). Lateral trunk lean explains variation in dynamic knee joint load in patients with medial compartment knee osteoarthritis. Osteoarthr. Cartil..

[25] Harato K (2008). Knee flexion contracture will lead to mechanical overload in both limbs: A simulation study using gait analysis. Knee.

[26] Murata Y, Takahashi K, Yamagata M, Hanaoka E, Moriya H (2003). The knee-spine syndrome. Association between lumbar lordosis and extension of the knee. J. Bone Jt. Surg. Br..

[27] Yasuda T (2020). Relationship between knee osteoarthritis and spinopelvic sagittal alignment in volunteers over 50 years of age. Asian Spine J..

[28] Ohkoshi Y, Yasuda K, Kaneda K, Wada T, Yamanaka M (1991). Biomechanical analysis of rehabilitation in the standing position. Am. J. Sports Med..

[29] Slemenda C (1997). Quadriceps weakness and osteoarthritis of the knee. Ann. Intern. Med..

[30] Kim YM, Joo YB (2012). Patellofemoral osteoarthritis. Knee Surg. Relat. Res..

[31] Kim HJ (2006). Influences of trunk muscles on lumbar lordosis and sacral angle. Eur. Spine J..

[32] Sparrey CJ (2014). Etiology of lumbar lordosis and its pathophysiology: A review of the evolution of lumbar lordosis, and the mechanics and biology of lumbar degeneration. Neurosurg. Focus.

[33] Lee S, Kim TN, Kim SH (2014). Knee osteoarthritis is associated with increased prevalence of vertebral fractures despite high systemic bone mineral density: a cross-sectional study in an Asian population. Mod. Rheumatol..

[34] Brouwer RW, Jakma TS, Verhagen AP, Verhaar JA, Bierma-Zeinstra SM (2005). Braces and orthoses for treating osteoarthritis of the knee. Cochrane Database Syst. Rev..

[35] Latham N, Liu CJ (2010). Strength training in older adults: The benefits for osteoarthritis. Clin. Geriatr. Med..

[36] Chen WH, Tsai WC, Wang HT, Wang CH, Tseng YT (2019). Can early rehabilitation after osteoarthritis reduce knee and hip arthroplasty risk? A national representative cohort study. Medicine (Baltimore).

[37] Roos EM, Arden NK (2016). Strategies for the prevention of knee osteoarthritis. Nat. Rev. Rheumatol..

[38] Heidari B (2011). Knee osteoarthritis prevalence, risk factors, pathogenesis and features: Part I. Casp. J. Intern. Med..

[39] Alexander EJ, Andriacchi TP (2001). Correcting for deformation in skin-based marker systems. J. Biomech..

[40] Hatze H (2002). The fundamental problem of myoskeletal inverse dynamics and its implications. J. Biomech..

[41] Tang AC, Tang SF, Hong WH, Chen HC (2015). Kinetics features changes before and after intra-articular hyaluronic acid injections in patients with knee osteoarthritis. Clin. Neurol. Neurosurg..

[42] Ohmi T (2022). Differences in gait kinetics and kinematics between patients with rotating hinge knee and cruciate-retaining prostheses: A cross-sectional study. J. Phys. Ther. Sci..

